# Genetic markers of thrombophilia as predictors of outcome in colorectal cancer

**DOI:** 10.1007/s11239-025-03106-1

**Published:** 2025-05-27

**Authors:** Valéria Tavares, Catarina Lopes, Catarina Macedo-Silva, Mónica Farinha, João Costa, Maria Isabel Vilas-Boas, Sofia Pinelas, Joana Assis, Mário Dinis-Ribeiro, Deolinda Pereira, Carina Pereira, Rui Medeiros

**Affiliations:** 1 Molecular Oncology and Viral Pathology Group, Research Center of IPO Porto (CI-IPOP)/CI-IPOP@RISE (Health Research Network), Portuguese Oncology Institute of Porto (IPO Porto)/Pathology and Laboratory Medicine Department/Clinical Pathology/Porto Comprehensive Cancer Center Raquel Seruca (Porto.CCC), 4200-072 Porto, Portugal; 2 ICBAS – Instituto de Ciências Biomédicas Abel Salazar, Universidade do Porto, 4050-313 Porto, Portugal; 3 FMUP, Faculty of Medicine, University of Porto, 4200-072 Porto, Portugal; 4 Precancerous Lesions and Early Cancer Management Group, Research Center of IPO Porto (CI-IPOP)/CI-IPOP@RISE (Health Research Group), Portuguese Institute of Oncology of Porto (IPO Porto)/Porto Comprehensive Cancer Center Raquel Seruca (Porto.CCC), 4200-072 Porto, Portugal; 5 CINTESIS – Center for Health Technology and Services Research, University of Porto, 4200-450 Porto, Portugal; 6 Cancer Biology and Epigenetics Group, Research Center of IPO Porto (CI-IPOP)/CI-IPOP@RISE (Health Research Group)/Portuguese Oncology Institute of Porto (IPO Porto)/Porto Comprehensive Cancer Center Raquel Seruca (Porto.CCC), 4200-072 Porto, Portugal; 7 Pathology Department, Portuguese Institute of Oncology of Porto (IPOP), 4200-072 Porto, Portugal; 8 Oncology Department, Portuguese Institute of Oncology of Porto (IPOP), 4200-072 Porto, Portugal; 9 Clinical Research Unit, Research Center of IPO Porto (CI-IPOP)/CI-IPOP@RISE (Health Research Network), Portuguese Oncology Institute of Porto (IPO Porto)/Porto Comprehensive Cancer Center Raquel Seruca (Porto.CCC), 4200-072 Porto, Portugal; 10 CEBIMED, Faculty of Health Sciences, Fernando Pessoa University, 4200-150 Porto, Portugal; 11 Research Department, Portuguese League Against Cancer (NRNorte), 4200-172 Porto, Portugal

**Keywords:** Colorectal neoplasms, Haemostasis, Inflammation, Genetic markers, Polymorphism, Single nucleotide

## Abstract

Colorectal cancer (CRC) is the second leading cause of malignancy-related death worldwide, representing a significant health concern. Understanding the disease pathogenesis and identifying potential prognostic biomarkers is critical for improving patients’ clinical outcomes. Haemostatic components implicated in cancer-associated thrombosis (CAT) seem to favour CRC progression. As such, genetic markers of thrombophilia might be potential prognostic biomarkers among patients with this malignant disease. To offer perspectives, a retrospective cohort study with 204 CRC patients was conducted to investigate the impact of seven germline haemostatic gene determinants on patient prognosis. A sex-stratified analysis was performed as the variants seem to have a distinct influence depending on the patient’s sex. Genomic DNA was extracted from FFPE samples enriched in tumour cells. While the polymorphisms *CNTN6* rs6764623 (CC/CA vs. AA; adjusted hazard ratio (aHR) = 0.44; 95% confidence interval (CI), 0.20–0.96; *P* = 0.040), *PTGS2* rs20417 (GG vs. CC/CG; aHR = 2.88; 95%CI, 1.10–7.51; *P* = 0.031) and *RGS7* rs2502448 (TT vs. CT/CC; aHR = 2.35; 95%CI, 1.20–4.61; *P* = 0.013) were associated with the five-year risk of cancer recurrence, *ITGB3* rs5918 was a predictor of the risk of death due to all causes, particularly among male patients (TT vs. CT/CC; aHR = 2.05; 95% confidence interval (CI), 1.13–3.72; *P* = 0.019). While a sex-specific impact of the SNPs was observed, further investigation in larger cohorts, particularly with an increased representation of female patients, is required to confirm these associations. Collectively, these markers could help improve the prognosis assessment of CRC patients towards a more personalised intervention.

**Graphical abstract:**

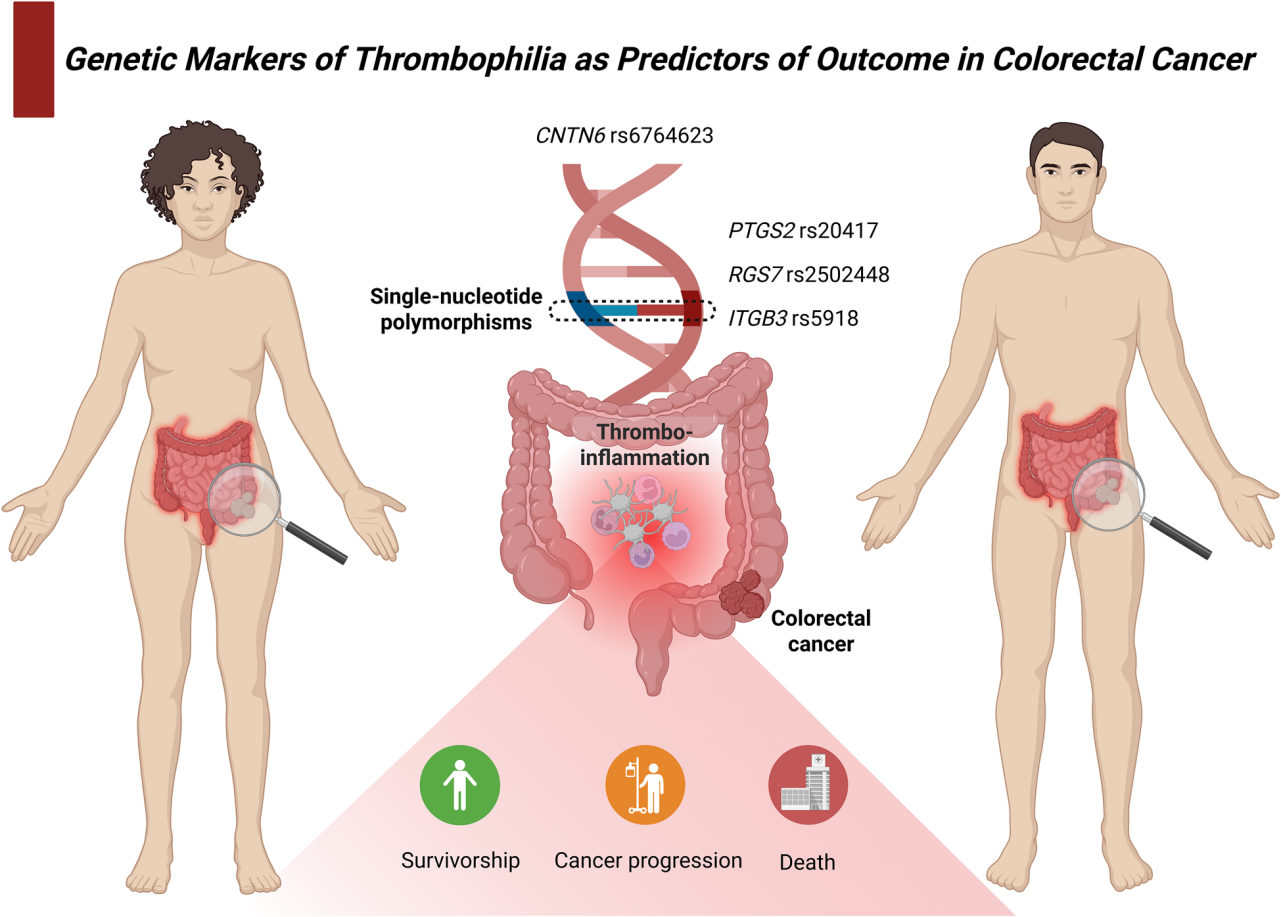

**Supplementary Information:**

The online version contains supplementary material available at 10.1007/s11239-025-03106-1.

## Highlights

- Despite major advances, the prognosis of CRC remains poor.
- Deregulated haemostatic mechanisms fuel colorectal carcinogenesis.
- Haemostasis-related genetic variants could be prognostic biomarkers of CRC.

## Introduction

Colorectal cancer (CRC) is the third most common malignancy and the second leading cause of cancer-related death worldwide, with reports of 1.93 million new cases and around 904,000 deaths in 2022 [1]. Despite major advances in disease treatment, the prognosis associated with CRC remains poor [2]. In addition to better treatment approaches, more prognostic factors are still to be identified, with over 20% of CRC patients thought to have an improper prognosis assessment [3].

In the era of personalised medicine, the need for predictive and prognostic biomarkers with clinical applications and proven benefits is increasing [4]. Innovations in tumour biology, including multi-omics approaches, have led to the identification of several candidate biomarkers. However, few of them have been effectively implemented in clinical practice [5]. Meanwhile, haemostasis represents an attractive source of tumour biomarkers [6]. Haemostasis is a protective biological process that allows the blood to circulate continuously. Haemostatic abnormalities can lead to pathological states of blood hypocoagulability (i.e., haemorrhage) and hypercoagulability (i.e., thrombosis), both life-threatening conditions. Cancer-associated thrombosis (CAT) is a common complication among oncological patients, representing their second cause of death [7]. Furthermore, deregulated haemostatic components presented in tumour microenvironments—platelets, endothelial cells and plasma proteins of the coagulation and fibrinolytic systems—can drive cancer growth and progression while increasing the risk of thrombotic events such as venous thromboembolism (VTE) [4, 8].

Among other leading diagnosed solid tumours (lung, breast and prostate cancers), CRC is associated with the second-highest CAT incidence [9, 10]. Indeed, haemostatic alterations are thought to be essential for the tumour dissemination [11]. Previous studies indicate that factor VII, D-dimer, prothrombin fragment 1 + 2 (F1 + 2) and fibrinogen could be valuable predictors of poor prognosis among CRC patients [11–13]. Furthermore, haemostasis-related genetic polymorphisms, particularly single-nucleotide polymorphisms (SNPs), have been associated with susceptibility for both CRC and CRC-related VTE, and are also linked to patient prognosis even in the absence of venous thrombogenesis [14]. Importantly, sex hormones (e.g., oestrogen, progesterone and testosterone) might also have a role in this interface between VTE and CRC. These hormones are recognised to influence haemostasis, modulating the risk for thrombotic events and potentially affecting the disease’s aggressiveness [15, 16]. Hence, the present study explored the implications of relevant SNPs on CRC patients’ clinical outcomes according to the patients’ sex.

## Materials and methods

### Patients

Patients of European ancestry with histologically diagnosed colorectal adenocarcinoma between January 2010 and December 2012, admitted to the Portuguese Institute of Oncology, Porto, Portugal (IPO Porto), were enrolled in a retrospective cohort study. These patients were consecutively selected based on the availability of formalin-fixed paraffin-embedded (FFPE) samples, after reviewing the histopathological data provided by the Pathology Department at IPO Porto. After excluding those with hereditary cancer, 204 patients were included in the study. Their medical records were reviewed to collect data regarding demographic and clinicopathological factors and follow-up. Tumour staging was performed using the TNM (Tumour Node Metastasis) staging system.

### Sample collection and genomic DNA extraction

Patient DNA was extracted from six slides (10 µm of thickness) containing up to 6 cm^2^ of macrodissected areas of FFPE samples enriched in tumour cells, using the AllPrep® DNA/RNA FFPE kit (Qiagen, Hilden, Germany), according to the manufacturer’s instructions. The concentration and purity of DNA samples were assessed spectrophotometrically (NanoDrop Lite spectrophotometer, Thermo Scientific®, Waltham, MA, USA).

The use of DNA isolated from FFPE samples of tumoural mucosa was validated by comparing it with DNA isolated from FFPE samples of normal mucosa of the same patient (n = 20/histology) using five tagSNPs previously characterised by our group, namely rs5275, rs20417 and rs689466 located within *prostaglandin-endoperoxide synthase 2* (*PTGS2*) and rs2555639 and rs2612656 located in or close to the *15-hydroxyprostaglandin dehydrogenase* (*HPGD*) gene [17]. Allelic discrimination was conducted via real-time polymerase chain reaction (qPCR) using validated TaqMan® SNP genotyping assays [C___7550203_10 for rs5275, C__11997909_40 for rs20417, C___2517145_20 for rs689466, C__16038735_10 for rs2555639 and C__15909858_20 for rs2612656 (Thermo Fisher Scientific, Waltham, MA, USA)].

### Polymorphism selection and genotyping

To cover different aspects of CAT, SNP selection was based on studies reporting genetic markers related to platelet activity and variants associated with VTE in the general population and with a prognostic value among oncological patients, independent of thrombotic events [14, 18, 19]. Furthermore, a minor allele frequency (MAF) of at least 10% in the Iberian population (*Ensembl* database, https://www.ensembl.org/index.html; accessed on 28 March 2022) was considered to avoid missing polymorphism genotypes in the cohort. Following these criteria, seven germline genetic polymorphisms were selected: rs4734879 in *zinc finger protein, FOG family member 2* (*ZFPM2*), rs2038024 in *solute carrier family 19 member 2* (*SLC19 A2*), rs6764623 in *contactin 6* (*CNTN6),* rs5918 in *integrin subunit beta 3* (*ITGB3)*, rs20417 in *PTGS2*, rs2502448 in *regulator of G protein signalling 7* (*RGS7*) and rs3779647 in *glutathione-disulfide reductase* (*GSR*).

Polymorphism genotyping using the patients’ DNA samples was conducted by the Centro Nacional de Genotipado at the Universidade de Santiago de Compostela, Spain, using MassARRAY® iPLEX Gold Technology (Agena Bioscience, San Diego, CA, USA). This method is based on multiplex amplification followed by mass-spectrometric product separation. The DNA samples had a concentration equal to or greater than 20 ng/µL. Of the 204 samples, four were excluded due to genotyping failure.

### Statistical analysis

Data analysis was conducted using IBM® SPSS® Statistics for Windows™ (version 25.0, SPSS Inc, 2016).

Associations of the genetic polymorphisms with patients’ demographic and clinicopathological factors (categorical variables) were evaluated using the chi-square test (χ^2^) or Fisher’s exact test.

Five-year disease-free survival (DFS) and overall survival (OS) were the clinical outcome measures. The former was defined as the time between patient diagnosis and either the date of disease recurrence or last follow-up in patients with complete response to first-line treatment. The latter was deemed the interval between patient diagnosis and death by any cause or last clinical evaluation. The endpoint definition was based on RECIST criteria (version 1.1) [20].

Survival curves were obtained using the Kaplan–Meier method, with survival probabilities analysed by the log-rank test when the proportional hazards assumption was met, or the Tarone-Ware test if not. The cumulative incidences of disease recurrence and mortality for the relevant SNPs across the entire cohort are presented in Supplementary Table S1. After an initial comparison between Kaplan–Meier curves under the additive genetic model, the most appropriate genetic model for each variant was chosen. Stratified analyses considering cancer stage (Supplementary Table S2) and sex were conducted for the relevant genetic polymorphisms (*P* < 0.05). The most adequate model in terms of cancer stages (III/IV vs. I/II or I/II/III vs. IV) was selected after comparing the Kaplan–Meier curves. To assess the risk of event over time, the five-year risk of cancer recurrence and the risk of death associated with the relevant SNPs were estimated using the Cox proportional hazards model. Multiple variable analyses using the backward stepwise (Wald) selection method were conducted, adjusting the impact of the relevant polymorphisms for demographic and clinicopathological factors with individual prognostic value according to univariate analyses (Supplementary Tables S3 and S4). All tests conducted were two-sided with a 5% level of significance.

## Results

### Population description

The mean follow-up was 79.2 ± 3.7 months. Characteristics of the population by sex are described in Table 1. The patients’ median age was 71 years (minimum = 23 years; maximum = 95 years), and most of them were diagnosed at cancer stages I/II (60%). Cancer therapeutic management consisted mainly of surgery (96%), adjuvant chemotherapy (24%), neoadjuvant chemotherapy (3%), neoadjuvant chemoradiotherapy (6%), neoadjuvant radiotherapy (1%) and palliative chemotherapy (14%).

**Table 1:** Demographic and clinicopathological characteristics of female and male colorectal cancer (CRC) patients (n = 200)

Characteristic	Sex			*P*-Value
	Female (n = 80; 40.0%)	Male (n = 120; 60.0%)	Total (n = 200)	
Age*				
Median (years)	72.5	71.0	71.0	**-**
≤ 71 years	38 (47.5)	66 (55.0)	104	0.370
> 71 years	42 (52.5)	54 (45.0)	96	
Site of primary disease				
Colon	60 (75.0)	90 (75.0)	150	1.000
Rectum	20 (25.0)	30 (25.0)	50	
Location of colon cancer**				
Left (sigmoid descending)	37 (46.3)	60 (50.0)	97	0.584
Right (transverse ascendent)	23 (28.8)	29 (24.2)	52	
Cancer stage**				
I	14 (17.5)	21 (17.5)	35	0.652
II	31 (38.8)	53 (44.2)	84	
III	30 (37.5)	35 (29.2)	65	
IV	5 (6.3)	10 (8.3)	15	
Cancer grade**				
Unknown	2 (2.5)	0 (0.0)	2	0.273
Low	69 (86.3)	109 (90.8)	178	
Moderate	1 (1.3)	1 (0.8)	2	
High	1 (1.3)	2 (1.7)	3	
Adjuvant treatment**				
Yes	20 (25.0)	28 (23.3)	48	0.906
No	59 (73.8)	91 (75.8)	150	
Neoadjuvant treatment**				
Yes	5 (6.3)	14 (11.7)	19	0.335
No	75 (93.8)	105 (87.5)	180	

*: Categories defined based on the median value given its not normal distribution (Kolmogorov–Smirnov test, *P* < 0.05); **: Number of subjects missing data: 1 for location of colon cancer, 1 for cancer stage, 15 for cancer grade, 2 for adjuvant treatment and 1 for neoadjuvant treatment

### Genotype distribution

The genotype frequency of each polymorphism is given in Table 2. No significant associations were observed (*P* ≥ 0.05) between the different SNPs and patients’ demographic and clinicopathological factors, including sex (male vs. female), age (≤ 71 vs. > 71 years), cancer stage (I vs. II vs. III vs. IV), tumour primary site (colon vs. rectum), location of colon cancer (left vs. right), neoadjuvant (no vs. yes) and adjuvant treatment (no vs. yes). An exception, however, was observed for *ZFPM2* rs4734879 polymorphism and patients’ age (≤ 71 vs. > 71 years). Namely, there was a higher frequency of GG genotype carriers (AA vs. AG vs. GG; *P* = 0.017) among younger patients compared to older ones (15 and 3, respectively).

**Table 2:** Genotype distribution of the evaluated polymorphisms in the study cohort (n = 200)

Polymorphism	MAF in the Iberian population (MA) *	Genotype	n (%)	MAF in the study population (MA)
*ZFPM2* rs4734879 (A > G)	33.6% (G)	AA	106 (53.0)	27.9% (G)
		AG	75 (37.5)	
		GG	18 (9.0)	
*SLC19 A2* rs2038024 (A > C)	18.2% (C)	AA	130 (65.0)	19.3% (C)
		CA	63 (31.5)	
		CC	7 (3.5)	
*CNTN6* rs6764623 (A > C)	25.2% (C)	AA	127 (63.5)	20.5% (C)
		CA	64 (32.0)	
		CC	9 (4.5)	
*ITGB3* rs5918 (T > C)	13.6% (C)	TT	131 (65.5)	18.8% (C)
		CT	61 (30.5)	
		CC	7 (3.5)	
*PTGS2* rs20417 (C > G)	15.0% (G)	CC	129 (64.5)	20.5% (G)
		CG	57 (28.5)	
		GG	12 (6.0)	
*RGS7* rs2502448 (T > C)	37.9% (C)	TT	52 (26.0)	46.5% (C)
		CT	110 (55.0)	
		CC	38 (19.0)	
*GSR* rs3779647 (T > C)	46.7% (C) **	TT	51 (25.5)	48.8% (T)
		CT	93 (46.5)	
		CC	56 (28.0)	

Genotyping failures were observed for *ZFPM2* rs4734879 [n = 1 (0.5%)], *ITGB3* rs5918 [n = 1 (0.5%)] and *PTGS2* rs20417 [n = 2 (1.0%)]

*MAF* minor allele frequency, *MA* minor allele

*: According to the *Ensembl* database; **: Considering all populations, T is the minor allele, with a frequency of 40% according to the *Ensembl* database

### Impact of haemostasis-related genetic polymorphisms on five-year disease-free survival

In the overall cohort, *ZFPM2* rs4734879, *SLC19 A2* rs2038024, *ITGB3* rs5918 and *GSR* rs3779647 did not exhibit a significant impact on five-year DFS, regardless of genetic model (additive, recessive or dominant), sex (male vs. female) and cancer stage (III/IV vs. I/II or I/II/III vs. IV).

*CNTN6* rs6764623 significantly impacted the five-year DFS considering the dominant model (CC/CA vs. AA; Supplementary Table S1; log-rank test, *P* = 0.041, Supplementary Figure S1). Namely, patients with the C allele (minor allele) presented a higher DFS than AA genotype carriers (mean five-year DFS of 54.9 ± 1.7 months and 50.4 ± 1.8 months, respectively), suggesting a protective effect of the C allele. A stratified analysis considering either sex (data not shown) or cancer stage (Supplementary Table S2) did not reveal significant associations (log-rank test, *P* ≥ 0.05).

**Fig. 1:**
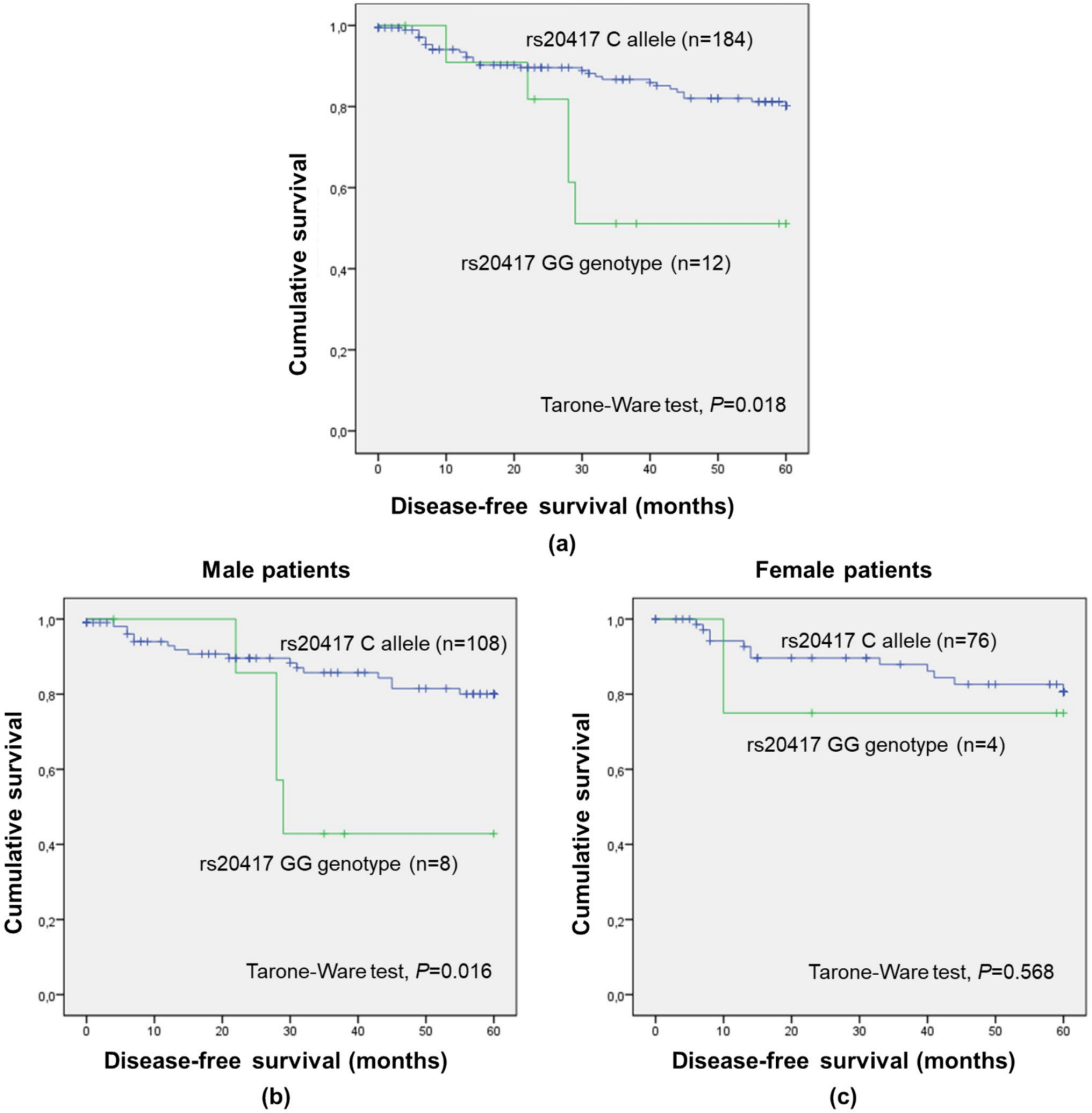
Five-year disease-free survival (DFS) by Kaplan–Meier and Tarone-Ware test for colorectal cancer (CRC) entire cohort (**a**; n = 196), male (**b**; n = 116) and female (**c**; n = 80) CRC patients, according to *PTGS2* rs20417 genotypes (recessive genetic model). **a** Patients with GG had lower five-year DFS than C allele carriers (mean five-year DFS of 42.3 months and 53.1 months, respectively; *P* = 0.018). **b** Male patients with the C allele had higher five-year DFS than GG genotype carriers (mean five-year DFS of 52.9 months and 41.0 months, respectively; *P* = 0.016). **c** No significant impact of the SNP among female patients was observed (*P* = 0.568)

Remarkably, a significant association was found between *PTGS2* rs20417 and five-year DFS considering the recessive model (GG vs. CC/CG; Supplementary Table S1; Tarone-Ware test, *P* = 0.018; Fig. 1a). Patients with the GG genotype (G being the minor allele) had lower DFS than C allele carriers (mean five-year DFS of 42.3 ± 5.9 months and 53.1 ± 1.3 months, respectively), indicating a detrimental impact of the GG genotype. When stratifying the analysis according to sex (Figs. 1b, c) and cancer stage (Supplementary Figures S2a and S2b), significant associations were observed only among male individuals (GG vs. CC/CG; Tarone-Ware test, *P* = 0.016; Fig. 1b) and patients with I/II stages (GG vs. CC/CG; Tarone-Ware test, *P* = 0.002; Supplementary Figure S2a).

**Fig. 2:**
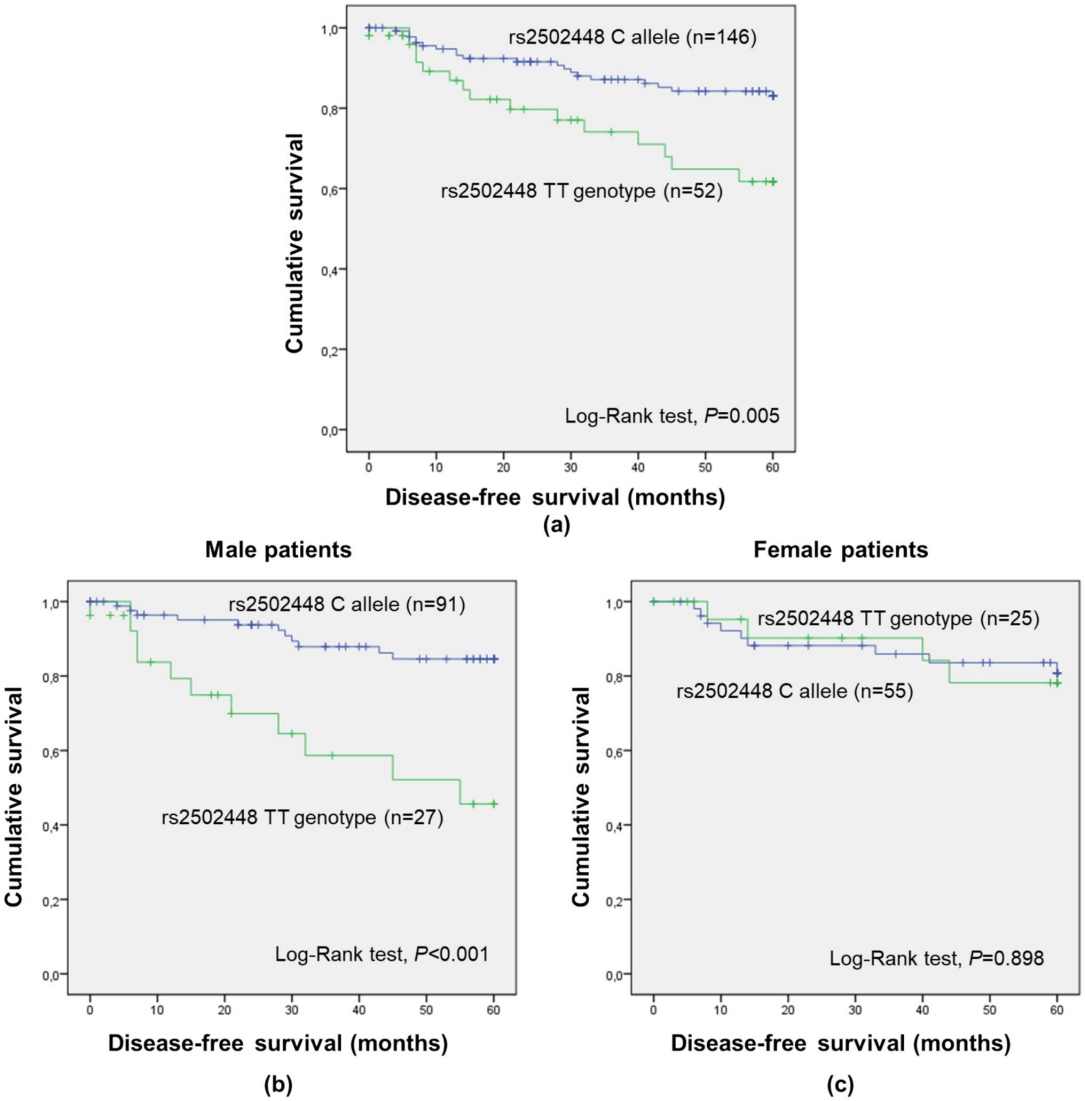
Five-year disease-free survival (DFS) by Kaplan–Meier and Log-rank test for colorectal cancer (CRC) entire cohort (**a**; n = 198), male (**b**; n = 118) and female (**c**; n = 80) patients, according to *RGS7* rs2502448 genotypes (dominant genetic model). **a** Patients with the C allele had higher five-year DFS compared to carriers of the TT genotype (mean five-year DFS of 54.0 months and 46.5 months, respectively; *P* = 0.005). **b** Male patients with the C allele had higher five-year DFS compared to TT genotype carriers (mean five-year DFS of 54.6 months and 40.4 months, respectively; *P* < 0.001). **c** No significant impact of the SNP among female patients was observed (*P* = 0.898)

Lastly, *RGS7* rs2502448 was found to have a significant impact on five-year DFS considering the dominant model (TT vs. CC/CT; Supplementary Table S1; log-rank test, *P* = 0.005; Fig. 2a). Patients with the C allele (minor allele) presented a prolonged DFS compared to TT genotype carriers (mean five-year DFS of 54.0 ± 1.4 months and 46.5 ± 3.1 months, respectively), linking the C allele to a protective effect. When stratifying the analysis considering patients’ sex (Figs. 2b, c) and cancer stage (Supplementary Figures S3a and S3b), significant associations were observed only among male individuals (TT vs. CC/CT; log-rank test, *P* < 0.001) and patients with III/IV stages (TT vs. CC/CT; log-rank test, *P* = 0.030; Supplementary Figure S3b).

Univariate Cox analyses corroborated the impact of *CNTN6* rs6764623, *PTGS2* rs20417 and *RGS7* rs2502448 on the five-year risk of disease recurrence in the entire cohort (Table 3).

**Table 3:** Impact of *CNTN6* rs6764623, *PTGS2* rs20417 and *RGS7* rs2502448 polymorphism on five-year risk of disease recurrence among colorectal cancer (CRC) patients, according to univariable Cox analysis

Variable	n	HR	95% CI	*P*-value
*CNTN6* rs6764623 (CC/CA vs. AA^1^)	198	0.45	0.21–0.99	0.048
*PTGS2* rs20417 (GG vs. CC/CG^1^)	196	3.19	1.23–8.30	0.017
*RGS7* rs2502448 (TT vs. CC/CT^1^)	198	2.54	1.30–4.96	0.006

*HR* hazard ratio, *CI* confidence interval, *N* number of patients included in the analysis

1: Reference group

Multivariable analyses using the backward stepwise selection method were carried out for each relevant polymorphism (Table 4) adjusted for the clinicopathological factors with individual prognostic value (*P* < 0.05) regarding the five-year risk of disease recurrence in our cohort of patients (Supplementary Table S3). Namely, cancer stage (III/IV vs. I/II), lymphovascular invasion (no vs. yes) and perineural invasion (no vs. yes). In addition, for *PTGS2* rs20417 and *RGS7* rs2502448, given the possible sex-specific effect of these polymorphisms, the variable sex was also included in the multivariable analyses. In the overall cohort, *CNTN6* rs6764623 was found to be an independent predictor of the five-year risk of disease recurrence (CC/CA vs. AA; adjusted HR (aHR) = 0.44; 95%CI, 0.20–0.96; *P* = 0.040). Specifically, C allele carriers had a 40% decrease in the risk of disease recurrence compared to patients with the AA genotype, adjusted for cancer stage. The polymorphism *PTGS2* rs20417 independently predicted the risk of disease recurrence both considering the entire cohort (GG vs. CC/CG; aHR = 2.88; 95%CI, 1.10–7.51; *P* = 0.031) or only male patients (GG vs. CC/CG; HR = 3.95; 95%CI, 1.31–11.97; *P* = 0.015; Table 4). Particularly, patients with the GG genotype have a three-to-fourfold increase in the risk of disease recurrence compared to C allele carriers. Likewise, *RGS7* rs2502448 was independently associated with the risk of cancer recurrence in the overall cohort (TT vs. CC/CT; aHR = 2.35; 95%CI, 1.20–4.61; *P* = 0.013) and among male patients (TT vs. CC/CT; aHR = 4.67; 95%CI, 2.00–10.88; *P* < 0.001; Table 4). Patients carrying the TT genotype have a two-to-fivefold increase in the risk of disease recurrence compared to C allele carriers.

**Table 4:** Impact of *CNTN6* rs6764623 (entire cohort, n = 198), *PTGS2* rs20417 (male patients, n = 116) and *RGS7* rs2502448 polymorphism (male patients, n = 118) on five-year risk of disease recurrence among colorectal cancer (CRC) patients, according to multivariable analysis (backward Wald method)

Variable	aHR	95% CI	*P*-value	aHR	95% CI	*P*-value
	Initial model			Final model *		
Lymphovascular invasion (no vs. yes^1^)	0.54	0.25–1.17	0.118	–	–	–
Perineural invasion (no vs. yes^1^)	0.54	0.25–1.19	0.127	–	–	–
Cancer stage (III/IV vs. I/II^1^)	1.85	0.90–3.83	0.095	2.54	**1.30–4.96**	**0.006**
*CNTN6* rs6764623 (CC/CA vs. AA^1^)	0.44	**0.20–0.98**	**0.045**	0.44	**0.20–0.96**	**0.040**

Variable	aHR	95% CI	*P*-value	aHR	95% CI	*P*-value
	Initial model			Final model *		
Cancer stage (III/IV vs. I/II^1^)	0.97	0.38–2.49	0.946	–	–	–
Lymphovascular invasion (no vs. yes^1^)	0.48	0.18–1.24	0.130	–	–	–
Perineural invasion (no vs. yes^1^)	0.88	0.27–2.91	0.837	–	–	–
*PTGS2* rs20417 (GG vs. CC/CG^1^)	3.15	0.95–10.47	0.061	3.95	**1.31–11.97**	**0.015**

Variable	aHR	95% CI	*P*-value	aHR	95% CI	*P*-value
	Initial model			Final model *		
Cancer stage (III/IV vs. I/II^1^)	0.99	0.38–2.62	0.986	–	–	–
Lymphovascular invasion (no vs. yes^1^)	0.39	0.15–1.03	0.056	0.39	**0.16–0.96**	**0.041**
Perineural invasion (no vs. yes^1^)	0.46	0.16–1.28	0.137	–	–	–
*RGS7* rs2502448 (TT vs. CC/CT^1^)	4.97	**2.11–11.69**	** < 0.001**	4.67	**2.00–10.88**	** < 0.001**

Bold values were considered statistically significant. 1: Reference group. *: After applying the backward Wald method

*aHR* adjusted hazard ratio, *CI* confidence interval

### Impact of haemostasis-related genetic polymorphisms on overall survival

Considering the entire cohort, for *ZFPM2* rs4734879, *SLC19 A2* rs2038024, *CNTN6* rs6764623, *PTGS2* rs20417, *RGS7* rs2502448 and *GSR* rs3779647, no significant impact on OS was detected, regardless of genetic model (additive, recessive and dominant), sex (male vs. female) and cancer stage (III/IV vs. I/II or I/II/III vs. IV).

**Fig. 3:**
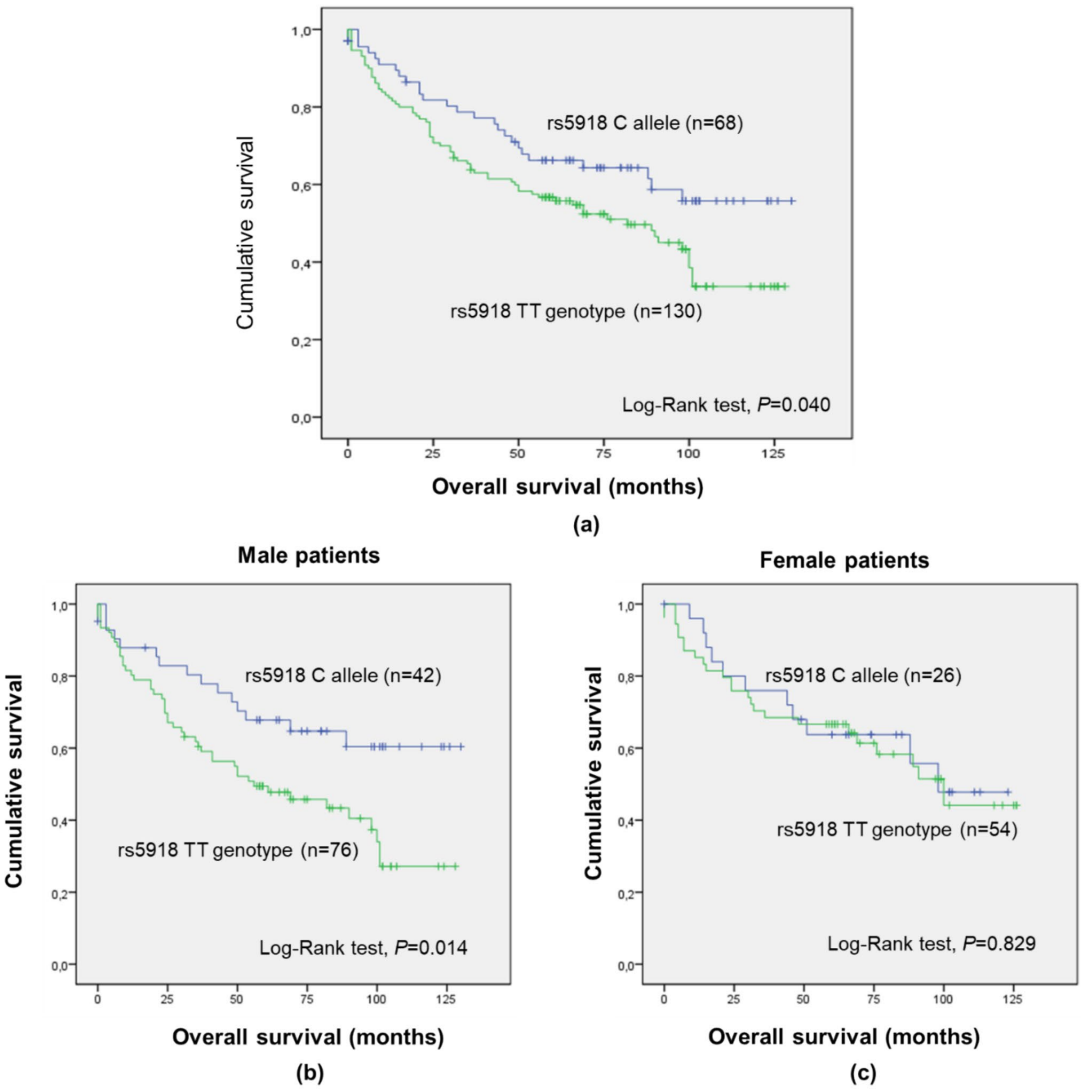
Overall survival (OS) by Kaplan–Meier and Log-rank test for colorectal cancer (CRC) entire cohort (**a**; n = 198), male (**b**; n = 118) and female (**c**; n = 80) patients, according to *ITGB3* rs5918 genotypes (dominant genetic model). **a** Patients with the C allele had higher OS compared to TT genotype carriers (mean OS of 90.5 months and 72.6 months, respectively; *P* = 0.040). **b** Male patients with the C allele had higher OS than TT genotype carriers (mean OS of 92.6 months and 66.3 months, respectively; *P* = 0.014). **c** No significant impact of the SNP among female patients was observed (*P* = 0.829)

As for *ITGB3* rs5918, a significant impact was observed considering the dominant model (TT vs. CC/CT; Supplementary Table S1; log-rank test, *P* = 0.040, Fig. 3a). Namely, patients with the C allele (minor allele) presented a mean OS of 90.5 ± 6.2 months, while TT genotype carriers exhibited a mean OS of 72.6 ± 4.5 months, suggesting a protective effect of the C allele. Considering the same genetic model and stratifying the analysis according to sex (male vs. female), the impact of the SNP was statistically significant among males (log-rank test, *P* = 0.014; Fig. 3b) but not among female patients (log-rank test, *P* = 0.829; Fig. 3c). Among male patients, C allele carriers had a longer OS than their counterparts (mean OS of 92.6 ± 7.9 months and 66.3 ± 5.8 months, respectively; Fig. 3b). Furthermore, by stratifying the analysis according to cancer stage (III/IV vs. I/II and I/II/III vs. IV), *ITGB3* rs5918 significantly impacted OS among patients without distant metastases (I/II/III vs. IV; log-rank test, *P* = 0.021; Supplementary Figure S4a) but not among their counterparts (log-rank test, *P* = 0.147; Supplementary Figure S4b). Namely, among patients with I/II/III disease stages, the ones with the C allele and TT genotype presented a mean survival time of 96.4 ± 6.1 months and 75.3 ± 4.8 months, respectively, corroborating a beneficial effect of the rs5918 C allele.

Univariate analyses corroborated the impact of *ITGB3* rs5918 on the risk of death in the entire cohort (TT vs. CT/CC; HR = 1.60; 95% confidence interval (CI), 1.02–2.50; *P* = 0.042). Multivariable analyses using the backward stepwise (Wald) selection method (Table 5) were conducted, adjusting for the effect of the polymorphism for the clinicopathological factors with individual prognostic value (*P* < 0.05) regarding the risk of death in the patient cohort (Supplementary Table S4). Namely, age (≤ 71 vs. > 71 years), cancer stage (III/IV vs. I/II or I/II/III vs. IV), lymphovascular invasion (no vs. yes), perineural invasion (no vs. yes) and adjuvant treatment (no vs. yes). No significant impact of sex on the risk of death was observed (*P* = 0.260; Supplementary Table S4), however, this variable was also included in the multivariable analyses, given the apparent sex-specific effect of rs5918 polymorphism observed in this study. In the overall cohort, no significant predictive impact of *ITGB3* rs5918 was observed in the multivariable analyses (*P* ≥ 0.05), regardless of whether sex was considered a variable. However, considering only male patients, rs5918 was an independent predictor of the risk of death. Namely, male patients with the rs5918 TT genotype had a twofold increase in the risk of death compared to their counterparts, adjusted for age, cancer stage, adjuvant treatment and lymphovascular invasion (TT vs. CT/CC; aHR = 2.05; 95%CI, 1.13–3.72; *P* = 0.019; Table 5).

**Table 5:** Impact of *ITGB3* rs5918 polymorphism on the risk of death among male colorectal cancer (CRC) patients (n = 118) according to multivariable analysis (the backward Wald method)

Variable	aHR	95% CI	*P*-value	aHR	95% CI	*P*-value
	Initial model			Final model*		
Age (≤ 71 vs. > 71 years^1^)	0.26	**0.14–0.48**	** < 0.001**	0.26	**0.15–0.48**	** < 0.001**
Cancer stage (I/II/III vs. IV^1^)	0.17	**0.08–0.38**	** < 0.001**	0.17	**0.08–0.38**	** < 0.001**
Adjuvant treatment (no vs. yes^1^)	1.80	0.89–3.63	0.100	1.81	0.90–3.64	0.097
Linfovascular invasion (no vs. yes^1^)	0.49	**0.28–0.85**	**0.011**	0.48	**0.28–0.84**	**0.010**
Perineural invasion (no vs. yes^1^)	0.96	0.51–1.84	0.911	–	–	–
*ITGB3* rs5918 (TT vs. CC/CT^1^)	2.04	**1.11–3.72**	**0.021**	2.05	**1.13–3.72**	**0.019**

Bold values were considered statistically significant. 1: Reference group. *: After applying the backward Wald method

*aHR* adjusted hazard ratio, *CI* confidence interval

## Discussion

Altered platelet activity and the overall perturbance of the physiological haemostatic balance are often observed among patients with malignant diseases [6]. Beyond VTE, which is a common cancer-related coagulopathology with a detrimental impact on the patient’s prognosis, microvascular thrombosis appears to be a critical event for tumour growth and dissemination [6, 8]. As CRC is one of the solid tumours most strongly associated with VTE development, this malignant disease is the perfect model to study how haemostatic components and the related genetic markers influence tumour progression [9, 10]. The present study was thus designed to explore the role of thrombosis-related SNPs in the clinical outcomes of CRC patients. As sex potentially influences the effect magnitude of these variants, a sex-stratified analysis was conducted [21]. Noteworthy, sex differences include both sexual dimorphism (alterations in genes and hormones) and gender differences (distinct behaviour and societal attitudes) [22]. In this study, no significant differences were observed in terms of demographic and clinicopathological characteristics between female and male patients, nor was sex shown to impact five-year DFS and OS. However, the influence of this variable cannot be dismissed. In fact, its effect on CRC patients’ survival might be dependent on other factors, namely age and hormonal status, which need to be assessed in future analyses [23].

Concerning five-year DFS, significant associations were found regarding the polymorphisms *CNTN6* rs6764623, *PTGS2* rs20417 and *RGS7* rs2502448. The variant rs6764623 (intergenic SNP and upstream transcript variant according to *Ensembl* and *NCBI* databases, respectively) is located close to *CNTN6* [24, 25]. This gene encodes a neural adhesion molecule that mediates cell surface interactions during nervous system development, acting also as a ligand for NOTCH1, triggering the Notch pathway. NOTCH1 is implicated in cardiovascular diseases and is thought to have a dual role in carcinogenesis, depending on the specific cellular context [6, 26, 27]. As for the SNP, rs6764623 is known to lead to an adenine-to-cytosine alteration, yet with no currently recognised functional impact [24]. Nevertheless, in a genome-wide association study (GWAS), the rs6764623 C allele was associated with an increased risk for VTE in the general population [relative risk (RR) = 1.18; *P* = 1.57 × 10^–6^] [28]. In the present study, the C allele was linked to a positive impact on the five-year DFS among CRC patients (log-rank test, *P* = 0.041), which was corroborated in univariate (*P* = 0.048) and multivariable Cox analyses (CC/CA vs. AA; aHR = 0.44; 95%CI, 0.20–0.96; *P* = 0.040). Specifically, CRC patients with the C allele had over 50% decrease in the risk of disease recurrence compared to carriers of the AA genotype. Although further investigation with well-defined larger cohorts is required, these contracting findings could be explained, for instance, by the dual roles of the Notch signalling depending on the cellular context and tumour microenvironment [29]. However, to the best of our knowledge, no suppressor role of Notch signalling, specifically NOTCH1, is described in CRC [30]. Furthermore, CNTN6 as a mediator of cell surface interactions may play additional tissue and context-specific roles that could explain these results. Lastly, no cancer stage and sex-specific impact of this polymorphism were observed in our cohort.

The SNP rs20417 (non-coding transcript exon SNP) is a variant located within the promoter of the *PTGS2* gene, which encodes a protein with the same name, also known as cyclooxygenase 2 (COX-2) [24, 31]. This protein is an inducible enzyme regulated by specific stimulatory events, which play roles in mitogenesis and inflammation via the conversion of arachidonic acid to prostaglandins, two pro-inflammatory mediators [32]. This enzyme may be crucial for platelet formation since it is upregulated in the late stages of megakaryopoiesis differentiation and characterises newly released thrombocytes, with evidence showing also a role in platelet aggregation and aspirin resistance [19, 33]. A large body of evidence implicates COX-2 in tumourigenesis, with most studies focusing on the role of the protein in cancer susceptibility, including CRC, which is considered an inflammation-driven malignancy. While COX-2 is not constitutively expressed in normal colon mucosa, over 90% of colon carcinomas overexpress this protein. In fact, COX-2 inhibition is found to reduce polyp formation [34]. Besides tumour development, COX-2 expression was also previously associated with an increased risk of CRC recurrence and risk of death [35]. The polymorphism rs20417 leads to a substitution of cytosine-to-guanine (most common) or cytosine-to-thymine [24]. Given the SNP location within the *PTGS2* promoter and its impact on platelet reactivity, the G allele (minor allele) is thought to be associated with a higher promoter activity, leading to increased protein levels. As such, this allele is potentially linked to a poor prognosis among oncological patients [36–38]. Consistently, in this study, the GG genotype was associated with reduced time to disease recurrence compared to C allele genotypes (Tarone-Ware test, *P* = 0.018). This finding was corroborated in univariate Cox analysis considering the overall cohort (*P* = 0.017). When stratifying the analysis in terms of disease stages, the association remained significant only among patients at early cancer stages (I/II). We hypothesised that the effect of this polymorphism might be relevant in a pre-metastatic phase, but once tumour dissemination occurs, other factors implicated in tumour progression might be more impacting. Interestingly, most haemostatic gene polymorphisms seem particularly relevant in early tumour stages [14]. Furthermore, considering separately female and male patients, rs20417 had a predictive impact only among male patients. Although further investigation is required, it has been postulated that sex may modulate the effect of genetic polymorphisms, with over 12% of autosomal expression quantitative trait loci acting in a sex-specific fashion [39]. In particular, sex-specific associations have been found between genetic risk profiles and cardiovascular diseases, such as coronary heart disease and VTE [40, 41]. In concordance, a previous study conducted by our research group demonstrated that haemostatic gene polymorphisms may have a distinct impact on female and male patients with thrombotic events [42]. Furthermore, the male reproductive system is under the influence of sex hormones [43, 44]. Likewise, these hormones also influence the gut microbiota composition and diversity [45]. Not dismissing the small cohort size, and considering the underrepresentation of female patients, the effect of *PTGS2* rs20417 on colorectal carcinogenesis might differ depending on the individuals sex. Multivariate analyses demonstrated at least a marginal association between this polymorphism and the risk of disease recurrence, considering or not sex as a variable in the overall cohort or only male patients. While the limited cohort size and inherent data variability are evident in the broad confidence intervals associated with the predictive power of this SNP, the findings of this study are supported by a strong biological plausibility.

The intronic SNP rs2502448, which leads to thymine-to-cytosine alteration, lies within the *RGS7*, a gene that encodes for a protein with the same name, which modulates G protein-coupled receptor (GPCR) signalling pathways by inhibiting signal transduction [24, 31]. The protein is thought to regulate platelet activity via central modulation of α2-adrenoreceptors and serotonin 2 A receptors, with *RGS7* rs2502448 being previously associated with aspirin resistance [46, 47]. In the setting of cancer, beyond platelet activity, the role of RGS7 is not clear. However, the protein expression appears to be under the influence of tumour necrosis factor-α (TNF-α), a major inflammatory cytokine implicated in many human cancers [48]. In this study, the SNP C allele presented a beneficial impact on the five-year DFS compared to the TT genotype (log-rank test, *P* = 0.005), which was corroborated in the univariate Cox analysis (*P* = 0.006). Upon stratification, *RGS7* polymorphism had a greater impact in advanced stages, particularly in stage III. However, as little is known about the role of RGS7 in tumourigenesis, the biological mechanisms underlying this finding remain unexplored. Furthermore, *RGS7* rs2502448 had a predictive impact only among male patients. According to *The Human Protein Atlas* (https://www.proteinatlas.org/ accessed on 08 February 2024), male individuals may express high levels of RGS7, with a marked expression in the testis. However, it remains to be explored whether this result is a consequence of any underlying biological reason or due to the small cohort size. In multivariate analyses, despite broad confidence intervals reflecting underlying data variability, *RGS7* rs2502448 remained significantly associated with five-year disease recurrence risk across all tested models – whether or not sex was included as a variable, and even when considering only male patients. Thus, in the present study, *RGS7* rs2502448 emerged as the most relevant predictor of the five-year risk of disease recurrence among CRC patients, representing an attractive prognostic biomarker with potential clinical applicability.

Concerning OS, *ITGB3* rs5918 (missense variant) was the only polymorphism in this study that was a predictor of the risk of death [24]. This SNP is located within *ITGB3*, which encodes for the integrin β3 subunit (also known as GP IIIa), a member of the integrin complex formed by glycoprotein IIb/IIIa receptors found at the platelet’s surface. Glycoprotein IIb/IIIa receptors bind to several ligands, including fibrinogen, being critical for platelet activation, aggregation and adhesion, and thus, haemostasis maintenance [19]. GP IIIa is implicated in several signalling pathways involved in tumour cell proliferation, migration and invasion [49]. Inclusively, it seems to be involved in the ROS-induced invasion and migration of CRC cells through mitogen-activated protein kinase (MAPK) signalling [50]. Regarding the polymorphism, rs5918 is defined as a thymidine-to-cytosine alteration in the exon 2 (T176 C), inducing a leucine (PIA1)-to-proline (PIA2) substitution at amino acid 33 of GP IIIa. This alteration has implications for the protein’s three-dimensional configuration, influencing its affinity to fibrinogen [51, 52]. Specifically, the PlA2 allele (C allele) was previously associated with an increased affinity of GP IIIa to fibrinogen and platelet aggregability, as well as to aspirin resistance [19, 52, 53]. In opposition, in some studies, aspirin resistance was attributed to the PIA1 allele (T allele), or no significant association of the polymorphism with aspirin resistance or platelet reactivity was observed [19, 54]. Previous studies have, however, linked this variant to manifestations of venous (deep vein thrombosis) and arterial thromboembolism (coronary artery disease and ischemic stroke) [55–57]. In the present study, an association with OS was observed considering all cases (log-rank test, *P* = 0.040), which was confirmed by univariate Cox analysis (*P* = 0.042). When stratifying the analysis according to cancer stage (I/II/III vs. IV), the association remained statistically significant only among patients without distant metastasis (log-rank test, *P* = 0.021), suggesting a role of this SNP in a pre-metastatic phase. Concordantly, some studies have pinpointed that GP IIIa plays a key role in intracellular communication through extracellular vesicles, being crucial for tumour dissemination [58, 59]. The rs5918 SNP was also shown to impact OS only among male patients (log-rank test, *P* = 0.014), being an independent predictor of the risk of death in this subgroup (TT vs. CT/CC; aHR = 2.05; 95%CI, 1.13–3.72; *P* = 0.019). Consistently, some evidence supports a sex-hormonal regulation of GP IIIa expression and/or activation [60–63]. Additional studies with larger samples, particularly with more females, are required to investigate the impact of this polymorphism in female CRC patients. Overall, the present study’s findings suggest that the SNP C allele has a protective effect in terms of the risk of death among CRC patients. Indeed, the link between this variant and tumourigenesis has been intensively explored in different settings, including ovarian, breast and colorectal cancers [64–67]. Particularly, in CRC, Hofmann et al. (2011) [67] did not observe a significant association between this polymorphism and both DFS and OS. As some controversies surround the effect of this SNP, further investigation is warranted.

As for the polymorphisms *ZFPM2* rs4734879, *SLC19 A2* rs2038024 and *GSR* rs3779647, no significant associations were observed in this study. Briefly, *ZFPM2* rs4734879 (intronic SNP) and *SLC19 A2* rs2038024 (upstream gene SNP) are two polymorphisms previously associated with VTE susceptibility in GWAS [18]. As for *GSR* rs3779647 (intronic SNP), it is a variant suggested to modulate platelet reactivity [38, 47]. Additional studies should be conducted to investigate their impact on CRC prognosis in more depth.

In sum, the SNPs *CNTN6* rs6764623 (CC/CA vs. AA; aHR = 0.44; *P* = 0.040), *PTGS2* rs20417 (GG vs. CC/CG; aHR = 2.88; *P* = 0.031) and *RGS7* rs2502448 (TT vs. CT/CC; aHR = 2.35; *P* = 0.013) were associated with the five-year risk of cancer recurrence, while *ITGB3* rs5918 was a predictor of the risk of death due to all causes, particularly among male patients (TT vs. CT/CC; aHR = 2.05; *P* = 0.019). A sex-specific impact of the SNPs was observed, which requires further investigation.

This study had some limitations, including its retrospective nature and the small cohort size, particularly concerning female patients and individuals with advanced disease stages (III/IV). The former limitation hindered the collection of information on some clinicopathological factors, as the status of tumour mutations, while the latter may have limited the statistical power to detect small effects. The small cohort and the existence of data variability, reflected in the wide confidence intervals observed, particularly for the predictive power of *PTGS2* rs20417 and *RGS7* rs2502448, further highlight the need for cautious data interpretation and future validation in larger, prospective cohorts. Nevertheless, the study findings are supported by a strong biological plausibility and are consistent with previous research.

## Conclusions

Prognostic biomarkers of CRC, particularly to assess the risk of disease recurrence, are needed to better define patient subgroups and provide a more personalised intervention [3, 68]. Conversely, thrombosis-related polymorphisms constitute attractive prognostic biomarkers for CRC, given the complex interplay between tumour cells and haemostatic components, favouring cancer growth and dissemination. The present study evaluated the role of seven SNPs linked to haemostasis in 200 CRC patients. Four genetic variants were associated with either the five-year DFS (*CNTN6* rs6764623, *PTGS2* rs20417 and *RGS7* rs2502448) or OS (*ITGB3* rs5918). In general, the impact of these genetic variants seems to be cancer stage-dependent, with some revealing also a sex-specific impact. Nonetheless, these results require further investigation with larger cohorts with appropriate representation of female patients. Additionally, functional studies are required to assess the biological plausibility of these findings. It is also mandatory to explore the biological mechanisms associated with these genetic variants, as it could lead to the identification of therapeutic targets.

## Supplementary Information

Below is the link to the electronic supplementary material.

Supplementary file1 (DOCX 439 KB)

## Data Availability

The data presented in this study are available on request from the corresponding author.
