# Supplementary material for: Genetic markers of thrombophilia as predictors of outcome in colorectal cancer
Source: J Thromb Thrombolysis. 2025 May 27;58(5):663–78. doi: 10.1007/s11239-025-03106-1 (PMC12149255; doi:10.1007/s11239-025-03106-1)
Supplement: Supplementary file 1 — Supplementary file1 (DOCX 439 KB) [file 11239_2025_3106_MOESM1_ESM.docx]

**Genetic Markers of Thrombophilia as Predictors of Outcome in Colorectal Cancer**

**Supplementary file**

**Supplementary Table S1** – Cumulative incidences of colorectal cancer (CRC) recurrence and mortality according to the relevant genetic models of *CNTN6* rs6764623, *PTGS2* rs20417, *RGS7* rs2502448, and *ITGB3* rs5918

| **SNP** | **Genotype** | **N**  **patients** | **5-year disease recurrence**  **N (%)** | **Death**  **N (%)** |
| --- | --- | --- | --- | --- |
| *CNTN6* rs6764623 | CC/CA | 72 | 8 (11.1) | - |
|  | AA | 126 | 27 (21.4) | - |
| *PTGS2* rs20417 | CC/CG | 184 | 29 (15.8) | - |
|  | GG | 12 | 5 (41.7) | - |
| *RGS7* rs2502448 | CC/CT | 146 | 20 (13.7) | - |
|  | TT | 52 | 15 (28.8) | - |
| *ITGB3* rs5918 | CC/CT | 68 | - | 26 (38.2) |
|  | TT | 130 | - | 70 (53.8) |

Abbreviations: N, number; SNP, single-nucleotide polymorphism.

**Supplementary Table S2** – Probability of survival by log-rank test concerning *CNTN6* rs6764623, *PTGS2* rs20417, *RGS7* rs2502448 and *ITGB3* rs5918 according to cancer stage (IV vs. I/II/III and III/IV vs. I/II)

| **Genetic polymorphism** | **Genotype** | **Cancer stage** | **N patients** | **Mean survival time (SD)**  **(months)** | ***P*-value** | **Chosen model** |
| --- | --- | --- | --- | --- | --- | --- |
| **5-year disease-free survival** | | | | | | |
| *CNTN6* rs6764623 | AA | I/II | 76 | 54.0 (1.7) | 0.244 |  |
|  | CC/CA |  | 43 | 56.8 (1.6) |  |  |
|  | AA | III/IV | 50 | 44.2 (3.7) | 0.062 |  |
|  | CC/CA |  | 29 | 52.3 (3.5) |  |  |
|  | AA | I/II/III | 117 | NA | 0.081 |  |
|  | CC/CA |  | 67 | NA |  |  |
|  | AA | IV | 9 | NA | 0.184 |  |
|  | CC/CA |  | 5 | NA |  |  |
| *PTGS2*  rs20417 | GG | I/II | 6 | 40.5 (7.4) | **0.002 *** | **+** |
|  | CC/CG |  | 112 | 55.8 (1.2) |  |  |
|  | GG | III/IV | 6 | 43.6 (9.3) | 0.583 * |  |
|  | CC/CG |  | 72 | 48.5 (2.7) |  |  |
|  | GG | I/II/III | 10 | 42.1 (6.7) | **0.019 *** |  |
|  | CC/CG |  | 172 | 53.1 (1.3) |  |  |
|  | GG | IV | 2 | 44.0 (11.3) | 0.987 * |  |
|  | CC/CG |  | 12 | 54.9 (6.9) |  |  |
| *RGS7* rs2502448 | TT | I/II | 25 | 53.6 (2.9) | 0.255 | **+** |
|  | CC/CT |  | 94 | 55.5 (1.3) |  |  |
|  | TT | III/IV ** | 27 | 39.5 (4.9) | **0.030** |  |
|  | CC/CT |  | 52 | 51.2 (3.0) |  |  |
|  | TT | I/II/III ** | 47 | 46.2 (3.3) | **0.003** |  |
|  | CC/CT |  | 137 | 54.2 (1.3) |  |  |
|  | TT | IV | 5 | 34.0 (1.7) | 0.940 |  |
|  | CC/CT |  | 9 | 53.0 (9.3) |  |  |
| **Overall survival** | | | | | | |
| *ITGB3*  rs5918 | TT | I/II | 74 | 85.8 (5.7) | 0.385 |  |
|  | CC/CT |  | 45 | 95.6 (7.1) |  |  |
|  | TT | III/IV | 56 | 54.7 (6.5) | 0.094 |  |
|  | CC/CT |  | 23 | 72.8 (10.3) |  |  |
|  | TT | I/II/III | 120 | 75.3 (4.8) | **0.021** | **+** |
|  | CC/CT |  | 63 | 96.4 (6.1) |  |  |
|  | TT | IV | 10 | 43.9 (12.3) | 0.147 |  |
|  | CC/CT |  | 5 | 20.2 (5.2) |  |  |

Bold values were considered statistically significant. * Tarone-Ware test. ** - When considering separately cancer stages I, II, III and IV, the polymorphism was significantly associated with the 5-year disease-free survival only among patients with III stage (TT vs. CT/CC; log-rank test, *P*=0.014), with individuals carrying the C allele presenting higher survival time than their counterparts (mean survival of 51.1 months and 37.7 months, respectively). This explains why a significant association was detected in patients within the groups I/II/III and III/IV. Abbreviations: N, number of patients included in the analysis; Na – Not determined; SD, standard deviation.

**Supplementary Table S3** - Univariate analyses on the 5-year risk of disease recurrence

| **Variable** | **N**  **patients** | **HR** | **95% CI** | ***P*-value** |
| --- | --- | --- | --- | --- |
| Sex  (Male vs. female^1^) | 198 | 1.26 | 0.63-2.50 | 0.509 |
| Age  (≤71 vs. >71 years^1^) | 198 | 0.72 | 0.37-1.39 | 0.322 |
| Cancer stage  (III/IV vs I/II^1^) | 198 | 2.47 | **1.27-4.82** | **0.008** |
| Cancer stage  (I/II/III vs. IV^1^) | 198 | 0.51 | 0.15-1.67 | 0.264 |
| Tumour primary site  (Colon vs. rectum^1^) | 198 | 0.89 | 0.42-1.91 | 0.769 |
| Location of colon cancer  (Right vs. left^1^) | 135 | 0.77 | 0.32-1.82 | 0.546 |
| Tumour grade  (Others vs. well differentiated^1^) | 169 | 21.38 | 0.01-60642.69 | 0.450 |
| Linfovascular invasion  (No vs. yes^1^) | 198 | 0.43 | **0.21-0.90** | **0.025** |
| Perineural invasion  (No vs. yes^1^) | 198 | 0.39 | **0.18-0.84** | **0.016** |
| Adjuvant treatment  (No vs. yes^1^) | 197 | 1.13 | 0.53-2.41 | 0.760 |
| Neoadjuvant treatment  (No vs. yes^1^) | 198 | 0.66 | 0.23-1.87 | 0.432 |

1 Reference group. Bold values were considered statistically significant.

Abbreviations: CI, confidence interval; HR, hazard ratio; N, number.

**Supplementary Table S4** - Univariate analyses on the risk of death from all causes

| **Variable** | **N**  **patients** | **HR** | **95% CI** | ***P*-value** |
| --- | --- | --- | --- | --- |
| Sex  (Male vs. female^1^) | 199 | 1.27 | 0.84-1.92 | 0.260 |
| Age  (≤71 vs. >71 years^1^) | 199 | 0.38 | **0.25-0.58** | **<0.001** |
| Cancer stage  (III/IV vs I/II^1^) | 199 | 2.03 | **1.36-3.02** | **0.001** |
| Cancer stage  (I/II/III vs. IV^1^) | 199 | 0.32 | **0.18-0.57** | **<0.001** |
| Tumour primary site  (Colon vs. rectum^1^) | 199 | 0.96 | 0.60-1.52 | 0.849 |
| Location of colon cancer  (Right vs. left^1^) | 148 | 1.56 | 0.98-2.51 | 0.063 |
| Tumour grade  (Others vs. well differentiated^1^) | 183 | 21.26 | 0.14-3195.45 | 0.232 |
| Linfovascular invasion  (No vs. yes^1^) | 199 | 0.48 | **0.31-0.74** | **0.001** |
| Perineural invasion  (No vs. yes^1^) | 199 | 0.59 | **0.36-0.96** | **0.034** |
| Adjuvant treatment  (No vs. yes^1^) | 198 | 2.26 | **1.30-3.93** | **0.004** |
| Neoadjuvant treatment  (No vs. yes^1^) | 199 | 0.67 | 0.36-1.25 | 0.208 |

1: Reference group. Bold values were considered statistically significant.

Abbreviations: CI, confidence interval; HR, hazard ratio; N, number.


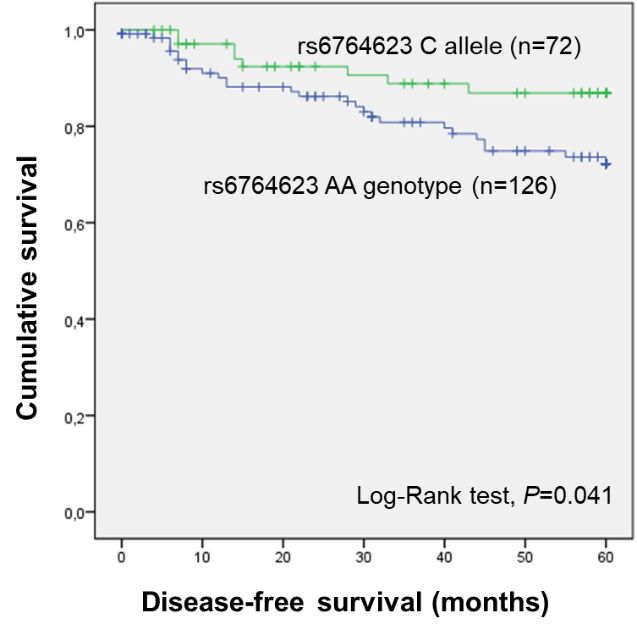


**Supplementary Figure S1** – Five-year disease-free survival (DFS) by Kaplan-Meier and Log-rank test for colorectal cancer (CRC) patients (n=198), according to *CNTN6* rs6764623 genotypes (dominant genetic model). Patients with the C allele had higher five-year DFS than carriers of the AA genotype (mean five-year DFS of 54.9 months and 50.4 months, respectively; *P*=0.041).


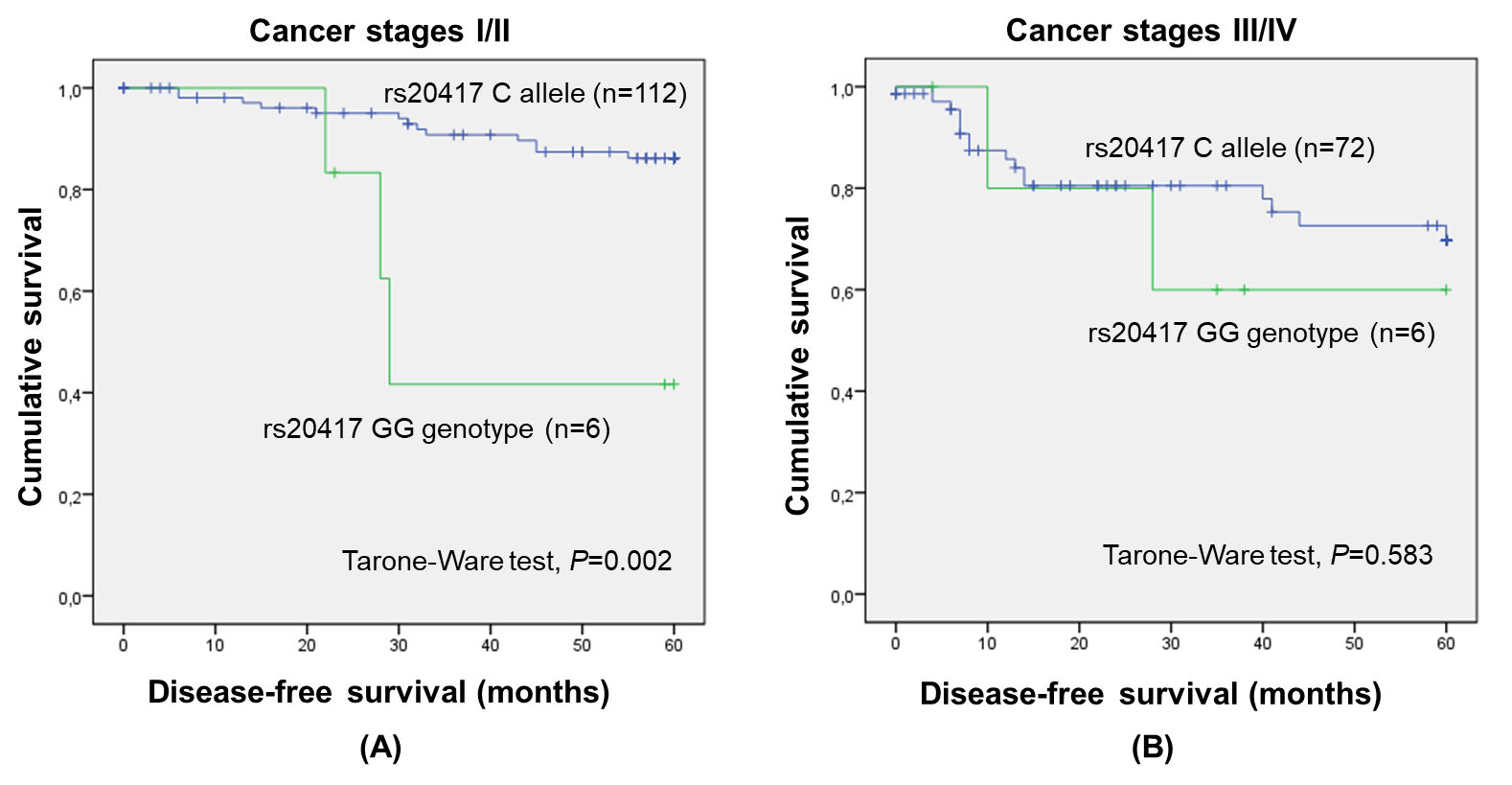


**Supplementary Figure S2** - 5-year disease-free survival (DFS) by Kaplan-Meier and Tarone-Ware test for colorectal cancer (CRC) patients at cancer stages I/II (**A**; n=118) and stages III/IV (**B**; n=78), according to *PTGS2* rs20417 genotypes (recessive genetic model). **A**) Patients at early disease stages (I/II) carrying the C allele had higher 5-year DFS compared to GG genotype carriers (mean 5-year DFS of 55.8 months and 40.5 months, respectively; *P*=0.002). **B**) No statistical association was observed among those at disease stages III/IV (*P*=0.583).


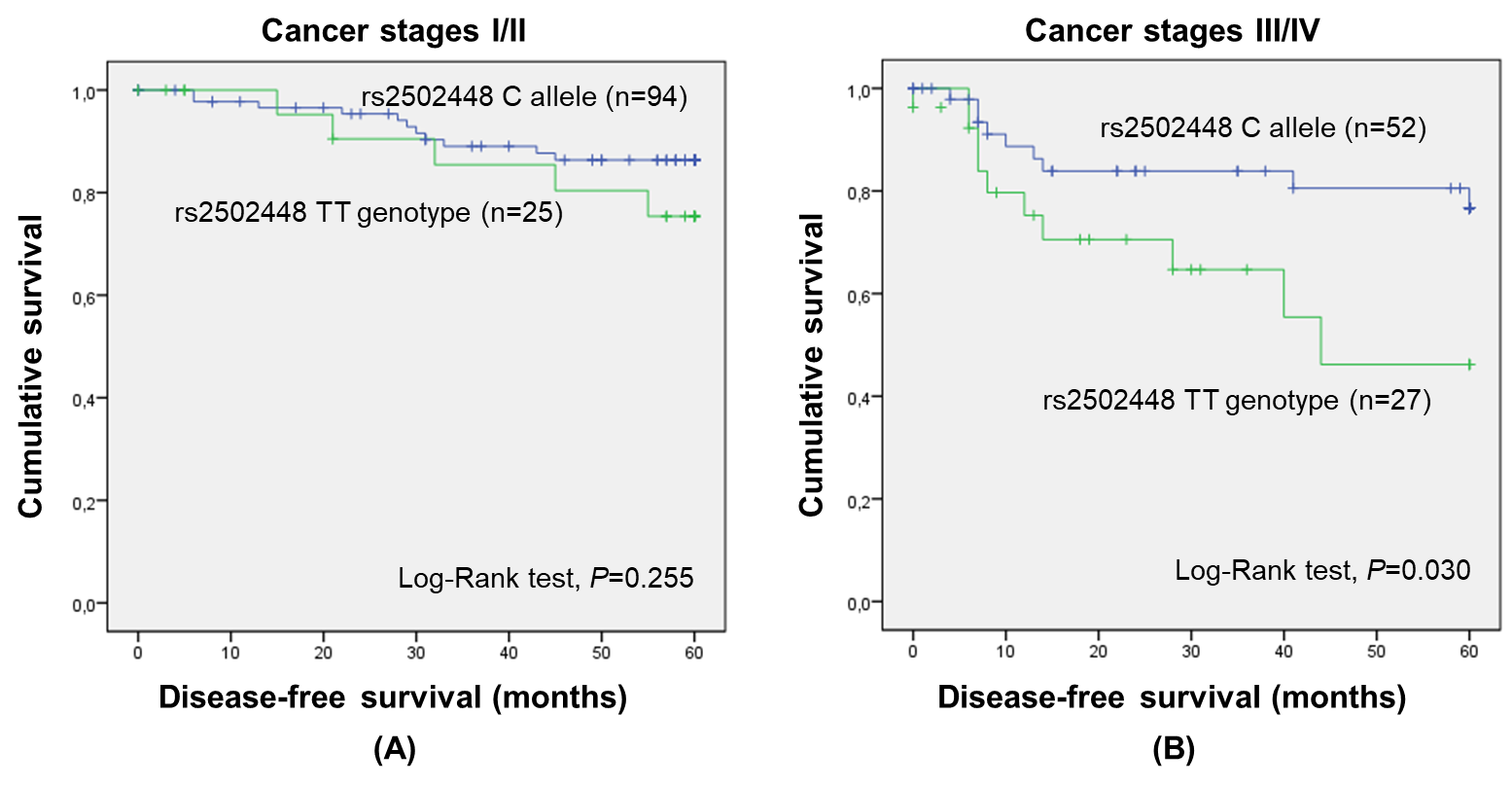


**Supplementary Figure S3** - 5-year disease-free survival (DFS) by Kaplan-Meier and Log-rank test for colorectal cancer (CRC) patients at cancer stages I/II (**A**; n=119) and stages III/IV (**B**; n=79), according to *RGS7* rs2502448 genotypes (dominant genetic model). **A**) No statistical association was observed among patients at disease stages I/II (*P*=0.255). **B**) Patients with advanced disease stages (III/IV) carrying the C allele had higher 5-year DFS compared to TT genotype carriers (mean 5-year DFS of 51.2 months and 39.5 months, respectively; *P*=0.030).


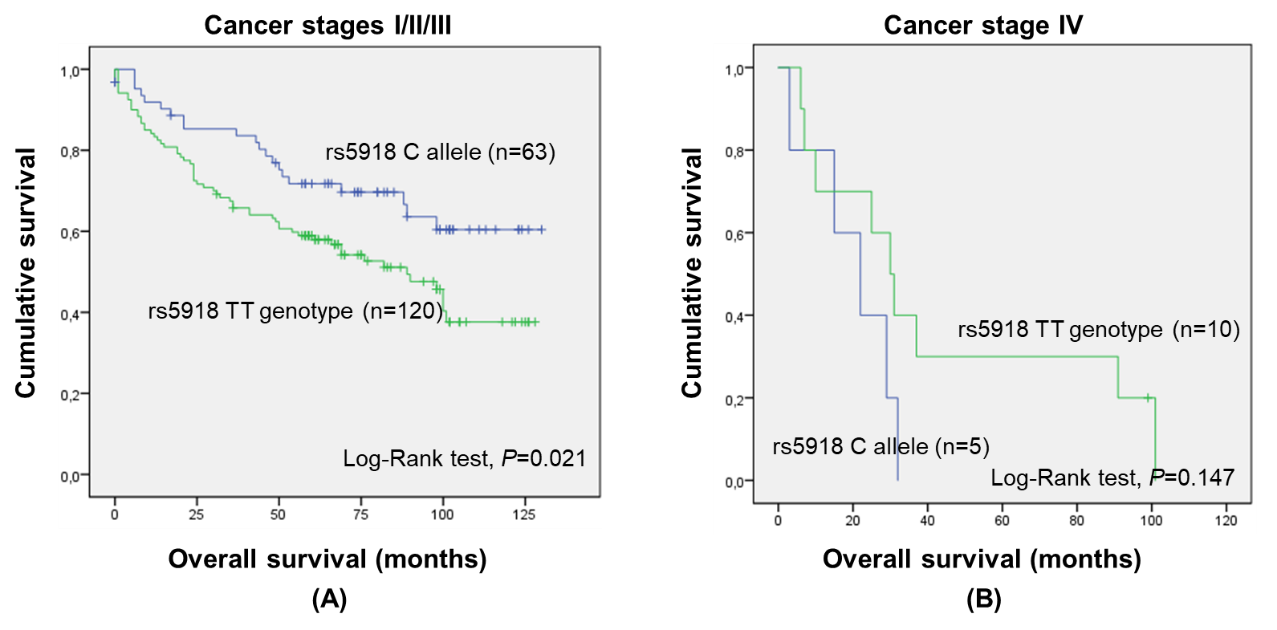


**Supplementary Figure S4** - Overall survival (OS) by Kaplan-Meier and Log-rank test for colorectal cancer (CRC) patients at cancer stages I/II/III (**A**; n=183) and stage IV (**B**; n=15), according to *ITGB3* rs5918 genotypes (dominant genetic model). **A**) Patients at stages I/II/III carrying the C allele had higher OS compared to TT genotype carriers (mean OS of 96.4 months and 75.3 months, respectively; *P*=0.021). **B**) No statistical association was observed among those at stage IV (*P*=0.147).
